# The Southern Branch of the National Dental Association

**Published:** 1898-04

**Authors:** 


### THE SOUTHERN BRANCH OF THE NATIONAL DENTAL ASSOCIATION.

This will, hereafter, be the name by which the Southern Dental Association will be known, at least this is the title as given by a correspondent of the *American Dental Weekly.*

The meeting was held at St. Augustine in February, and was, according to the same authority, a great success in point of numbers.

The papers presented were in excess of the time of three sessions a day and three days' work, and many were simply read by title.

Florida seems not to have come up to expectations as a State for conventions, for the correspondent remarks, "We are sure that the society has agreed unanimously that we will not go there again."

The success of this meeting, the first since the organization of the National Association, demonstrates the wisdom of continuing it as a branch and the folly of destroying the American Dental Association. This body could have been used as the Eastern Branch with equally good results.

An attempt is being made to organize a new body in the Eastern States, an effort involving great labor and an unnecessary waste of mental strength, but inasmuch as the past cannot be recalled, an organization similar to the Southern seems imperatively to be demanded.

